# Microbiology and prognostic prediction model of bloodstream infection in patients with hematological malignancies

**DOI:** 10.3389/fcimb.2023.1167638

**Published:** 2023-06-30

**Authors:** Jinjin Wang, Mengyao Wang, Ailin Zhao, Hui Zhou, Mingchun Mu, Xueting Liu, Ting Niu

**Affiliations:** ^1^ Department of Hematology, West China Hospital, Sichuan University, Chengdu, Sichuan, China; ^2^ Gastric Cancer Center, West China Hospital, Sichuan University, Chengdu, Sichuan, China; ^3^ Department of Medical Discipline Construction, West China Hospital, Sichuan University, Chengdu, Sichuan, China

**Keywords:** bloodstream infection, hematological malignancy, nomogram, model, microbiology, 30-day mortality, risk factor

## Abstract

**Background:**

In recent years, with the continuous development of treatments for hematological malignancies (HMs), the remission and survival rates of patients with HMs have been significantly improved. However, because of severe immunosuppression and long-term recurrent neutropenia during treatment, the incidence and mortality of bloodstream infection (BSI) were all high in patients with HMs. Therefore, we analyzed pathogens’ distribution and drug-resistance patterns and developed a nomogram for predicting 30-day mortality in patients with BSIs among HMs.

**Methods:**

In this retrospective study, 362 patients with positive blood cultures in HMs were included from June 2015 to June 2020 at West China Hospital of Sichuan University. They were randomly divided into the training cohort (n = 253) and the validation cohort (n = 109) by 7:3. A nomogram for predicting 30-day mortality after BSIs in patients with HMs was established based on the results of univariate and multivariate logistic regression. C-index, calibration plots, and decision curve analysis were used to evaluate the nomogram.

**Results:**

Among 362 patients with BSIs in HMs, the most common HM was acute myeloid leukemia (48.1%), and the most common pathogen of BSI was gram-negative bacteria (70.4%). The final nomogram included the septic shock, relapsed/refractory HM, albumin <30g/l, platelets <30×10^9^/l before BSI, and inappropriate empiric antibiotic treatment. In the training and validation cohorts, the C-indexes (0.870 and 0.825) and the calibration plots indicated that the nomogram had a good performance. The decision curves in both cohorts showed that the nomogram model for predicting 30-day mortality after BSI was more beneficial than all patients with BSIs or none with BSIs.

**Conclusion:**

In our study, gram-negative bacterial BSIs were predominant in patients with HMs. We developed and validated a nomogram with good predictive ability to help clinicians evaluate the prognosis of patients.

## Introduction

Hematological malignancies (HMs) are diseases originating from or implicating the hematologic system and hematopoietic tissue. The treatment of HMs includes chemotherapy, immunotherapy, targeted therapy, and hematopoietic stem cell transplantation (Auberger et al., 2020). In recent years, with the continuous development of treatments for HMs, the remission and survival rates of patients with HMs have significantly improved. However, because of severe immunosuppression and long-term recurrent neutropenia during treatment, the incidence (20.8%-24.1%) and mortality (10%-32%) of bloodstream infection (BSI) were all high in patients with HMs (Nørgaard et al., 2006; Åttman et al., 2015; Trecarichi et al., 2015; Tang et al., 2018; Chen et al., 2021; Okamoto et al., 2021; Zhao et al., 2022). Meanwhile, compared with solid tumors, patients with HMs had a higher risk for BSIs (Marin et al., 2014). A 14-year longitudinal surveillance study showed that the incidence of BSIs in patients with HMs was three times higher than that of other cancer (Schelenz et al., 2013).

BSI has many unfavorable effects on patients with HMs, such as the increase in mortality, the addition of medical bills, and the prolongation of the hospitalization period (Liao et al., 2021). Therefore, timely and effective control of BSI is vital for patients with HMs. On the one hand, antibiotic therapy is crucial to patients with BSIs, and no other treatment can replace the efficacy of antibiotics. Doctors usually treat patients with an empiric antibiotic to control BSIs and then adjust the antibiotic based on the drug sensitivity test results, as it takes some time to isolate the pathogens by blood culture. Investigating pathogens’ distribution and drug-resistance patterns contribute to choosing the appropriate empiric antibiotics (Kadri et al., 2021).

On the other hand, it was essential to identify the risk factors for mortality in patients with BSIs, and then conduct early intervention and management (Timsit et al., 2020). Studies have explored the risk factors of prognosis for patients with BSIs in hematological diseases (Tumbarello et al., 2012; Tang et al., 2018; Albasanz-Puig et al., 2021; Shi et al., 2022; Zhao et al., 2022). These risk factors included age >60, relapsed or uncontrol malignancies, nosocomial infection, prolonged neutropenia, profound neutropenia, inappropriate empiric antibiotics, albumin <30g/l, BSI with multiple drug-resistant (MDR) bacteria, placement of the central venous catheter (CVC), placement of the urinary catheter, decreased white blood cell, and so on. Many prediction models were applied to assess outcomes for patients at high risk of various diseases (Woo et al., 2016; Yang et al., 2020; Asami et al., 2022; Zhu et al., 2022). Nevertheless, there were few models for the prognosis of patients with BSIs in HMs (Tang et al., 2018; Zhao et al., 2022). Meanwhile, these models may not apply to patients with BSIs in this region due to regional differences.

West China Hospital of Sichuan University is one of the largest diagnosis and treatment centers for difficult miscellaneous diseases in Western China, and its data are representative. Therefore, based on data from our hospital, we analyzed pathogens’ distribution and drug-resistance patterns and established a nomogram model for predicting 30-day mortality in patients with BSIs among HMs, to provide evidence for the prevention and treatment of patients with BSIs in HMs.

## Methods

### Setting and study design

This retrospective study was conducted at the hematology department (215 beds) of West China Hospital, a national center for diagnosing and treating complex and critical diseases in Western China. The subjects in this study covered patients with positive blood cultures (bacteria or fungi) in HMs from June 2015 to June 2020. West China Hospital of Sichuan University Ethics Committee approved this study and waived informed consent because this study was retrospectively designed. We followed the following inclusion criteria to screen patients: (1) positive blood culture; (2) patient diagnosed with HM; (3) complete clinical data. The exclusion criteria were as follows: (1) patient diagnosed with non-HM; (2) incomplete clinical data; (3) patient with multiple positive blood cultures with the same pathogen during the same hospital stay; they were counted once.

### Data collection

Data were extracted from the Hospital Information System (HIS) of West China Hospital of Sichuan University. The details were as follows: ID number, gender, age, temperature, pathogens of BSIs, results of drug sensitivity test, blood pressure, state of disease, hematopoietic stem cell transplantation, nosocomial infection, length of stay before BSI, concurrent infection, white blood cell count, neutrophil count, platelet count, albumin level, procalcitonin (PCT), C-reactive protein (CRP), prothrombin time, activated partial thromboplastin time, D-dimer, invasive operation (urinary catheter, CVC), or peripherally inserted central catheter (PICC)), empiric antibiotic therapy, chemotherapy regimen, and outcomes at 30-day after BSI.

### Definitions

Patients with positive blood culture (excluding pollution) who had a fever (>38°C) and at the same time one or more symptoms, including chill, low oxygen saturation, hypotension, cold moist limbs, and disturbance of consciousness, were identified as true BSI (Amanati et al., 2021). Septic shock was defined as the vasopressor requirement for maintaining mean arterial pressure ≥65mmHg or serum lactate level ≥2mmol/l (Singer et al., 2016). Disseminated intravascular coagulation (DIC) was diagnosed with the Chinese DIC scoring system (Luo et al., 2019). MDR bacteria were defined as resistant to three or more antibacterial agents (Magiorakos et al., 2012). Empiric antibiotic therapy was defined as one or more antibiotics the patient received within 48 hours of being diagnosed with BSI (time to draw a positive blood culture specimen). Empiric antibiotic therapy was deemed appropriate if at least one of the empiric antibiotics was sensitive *in vitro* drug sensitivity test; otherwise, it was considered inappropriate empiric antibiotic therapy. Absolute neutrophil counts <0.5×10^9^/l and <0.1×10^9^/l were defined as neutropenia and profound neutropenia.

### Statistical analysis

A database was established with Excel, and statistical analysis was performed with IBM SPSS 26.0. All cases were randomly divided into a training cohort (n =253) and a validation cohort (n =109) by 7:3. Continuous variables were expressed with mean ± standard deviation or median (IQR) according to the distribution pattern, and t-test or Mann-Whitney U test was used to the comparison between two cohorts. Frequency and proportion were applied to represent classified variables, and the comparison between two cohorts was conducted by Chi-square test or Fisher’s exact test. The 30-day mortality risk factors were analyzed by binary logistic regression. Variables with p<0.05 in the univariate logistic regression analysis were included in the multivariate logistic regression analysis. Based on the result of multivariate logistic regression analysis, we used the package of rms in R version 4.1.1 (http://www.r-project.org/) to plot the nomogram. The C-index was used to evaluate the discrimination of the nomogram. If the C-index is larger, the discrimination of the nomogram model is better. The accuracy (the extent to which the nomogram predicted 30-day mortality in accordance with actual mortality) of the nomogram was assessed by calibration plots. Calibration at a 45° diagonal is best, and above or below this diagonal indicates under-prediction or over-prediction. Decision curve analysis (DCA) was conducted to evaluate the net clinical benefit by quantifying net benefits at different threshold probabilities.

## Results

### Patient characteristics

A total of 362 patients who had HMs with BSIs were included in the final analysis, 174 with acute myeloid leukemia, 74 with acute lymphocytic leukemia, 58 with non-Hodgkin lymphoma, and 56 with other HMs. All patients were randomly divided into the training cohort (n = 253) and the validation cohort (n = 109) by 7:3. The detailed characteristics of all patients are shown in **Table 1**. In all patients with an average age of 42.4 years, the male accounted for 64.6%, while the female accounted for 35.4%. The state of 108 patients with HMs was relapsed/refractory. A minority (6.9%) received hematopoietic stem cell transplantation, and a majority (86.7%) had concurrent pulmonary infections. Neutropenia existed in 79.3% of patients, and secondary DIC occurred in 20%. BSIs with MDR bacteria occurred in 204 patients. 35.1% of all patients received inappropriate empiric antibiotic therapy. 30-day mortality in patients with HMs after BSIs was 29%. Most variables were balanced between the training and validation cohorts, except for the PCT (p=0.002) (**Table 1**).

**Table 1:** Baseline characteristics of the training and validation cohorts.

Characteristic	All subjects (*n* = 362)	Training cohort (*n* = 253)	Validation cohort (*n* = 109)	*p* value
Sex, *n* (%)				1
Male	234 (64.6)	164 (64.8)	70 (64.2)	
Female	128 (35.4)	89 (35.2)	39 (35.8)	
Age, mean ± SD, years	42.4 ± 16.4	43.4 ± 16.1	40.1 ± 16.8	0.079
Pathogens, *n* (%)				0.348
Gram-negative bacteria	255 (70.4)	184 (72.7)	71 (65.1)	
Gram-positive bacteria	87 (24.0)	56 (22.1)	31 (28.4)	
Fungi	20 (5.5)	13 (5.1)	7 (6.4)	
Septic shock, *n* (%)	78 (21.5)	60 (23.7)	18 (16.5)	0.163
Relapsed/refractory HMs, *n* (%)	108 (29.8)	78 (30.8)	30 (27.5)	0.535
Hematopoietic stem cell transplantation, *n* (%)	25 (6.9)	20 (7.9)	5 (4.6)	0.273
Nosocomial infection, *n* (%)	326 (90.1)	226 (89.3)	100 (91.7)	0.568
Length of stay before BSI, median (IQR), days	18 (10,31)	19 (9, 29)	18 (11, 38)	0.495
Concurrent pulmonary infection, *n* (%)	314 (86.7)	224 (88.5)	90 (82.6)	0.131
Concurrent intestinal infection, *n* (%)	30 (8.3)	22 (8.7)	8 (7.3)	0.688
Concurrent perianal infection, *n* (%)	43 (11.9)	32 (12.6)	11 (10.1)	0.596
White blood cell count, median (IQR), 10^9^/l	0.42 (0.14, 2.86)	0.38 (0.13, 3.26)	0.54 (0.18, 2.4)	0.319
Neutropenia, *n* (%)	287 (79.3)	201 (79.4)	86 (78.9)	1
Profound neutropenia, *n* (%)	236 (65.2)	168 (66.4)	68 (62.4)	0.462
Albumin <30g/l, *n* (%)	115 (31.8)	86 (34.0)	29 (26.6)	0.178
PCT, median (IQR), ng/ml	0.79 (0.25, 6.3)	1.28 (0.29, 6.3)	0.49 (0.19, 2.52)	0.002
CRP, median (IQR), mg/l	82.47 (44.3, 123.11)	84.2 (50.08, 124.42)	76.4 (36.85, 121.03)	0.224
Platelets <30×10^9^/l before BSI, *n* (%)	240 (66.3)	172 (68.0)	68 (62.4)	0.333
Platelets <30×10^9^/l after BSI, *n* (%)	266 (73.5)	192 (75.9)	74 (67.9)	0.121
Placement of urinary catheter, *n* (%)	50 (13.8)	39 (15.4)	11 (10.1)	0.178
Placement of PICC/CVC, *n* (%)	174 (48.1)	122 (48.2)	52 (47.7)	1
Secondary DIC, *n* (%)	73 (20.2)	52 (20.6)	21 (19.3)	0.887
BSI with MDR bacteria, *n* (%)	204 (56.4)	143 (56.5)	61 (56.0)	1
Inappropriate empiric antibiotic treatment, *n* (%)	127 (35.1)	86 (34.0)	41 (37.6)	0.549
Combined empiric antibiotic treatment, *n* (%)	268 (74.0)	188 (74.3)	80 (73.4)	0.896
Chemotherapy regimen containing glucocorticoid, *n* (%)	142 (39.2)	101 (39.9)	41 (37.6)	0.726
30-day mortality, *n* (%)	105 (29)	72 (28.5)	33 (30.3)	0.801

### Microbiology

**Figure 1:**
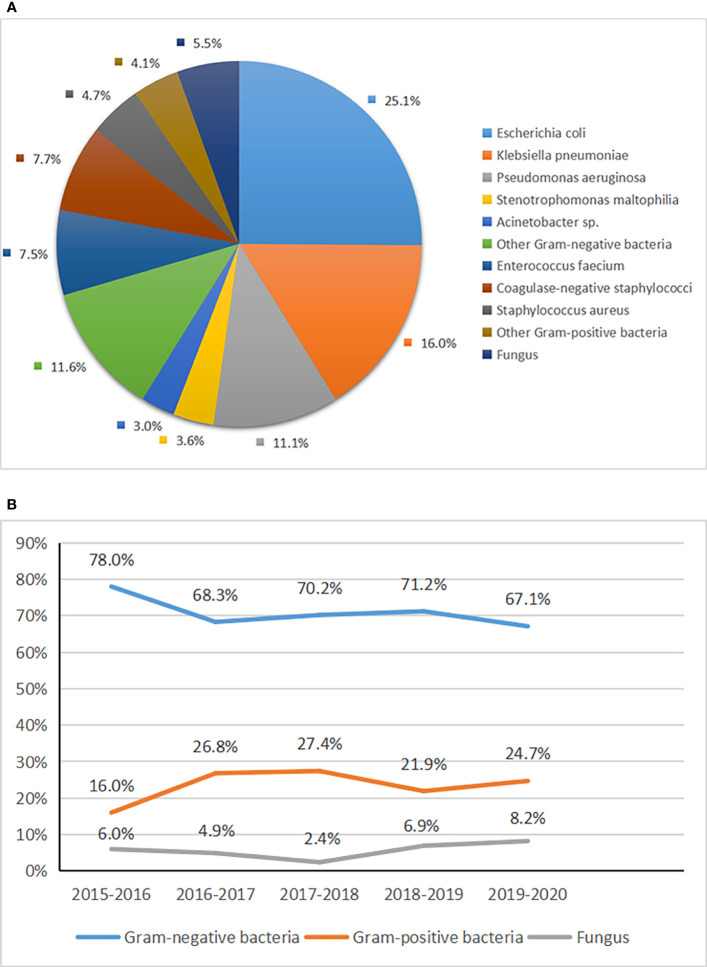
Distribution **(A)** and trend **(B)** in pathogens of BSIs among patients with HMs.

Three hundred sixty-two pathogens were isolated from blood culture, including gram-negative bacteria (70.4%), gram-positive bacteria (24%), and fungi (5.5%) (**Figure 1**). The most common gram-negative bacterium was *Escherichia coli*, accounting for 25.1%, followed by *Klebsiella pneumoniae* (16.0%) and *Pseudomonas aeruginosa* (11.1%). The common gram-positive bacteria were coagulase-negative staphylococci (7.7%), *Enterococcus faecium* (7.5%), and *Staphylococcus aureus* (4.7%). Seven strains of methicillin-resistant *Staphylococcus aureus* (MRSA) and 21 strains of methicillin-resistant coagulase-negative staphylococcus (MRCNS) were detected in all of *Staphylococcus aureus* and coagulase-negative staphylococci. The most common fungus was *Candida tropicalis*, accounting for about 2.8%. Although the proportion of gram-negative bacteria, gram-positive bacteria, and fungi fluctuated from 2015 to 2020, gram-negative bacteria were still dominant (**Figure 1**).

**Figure 2:**
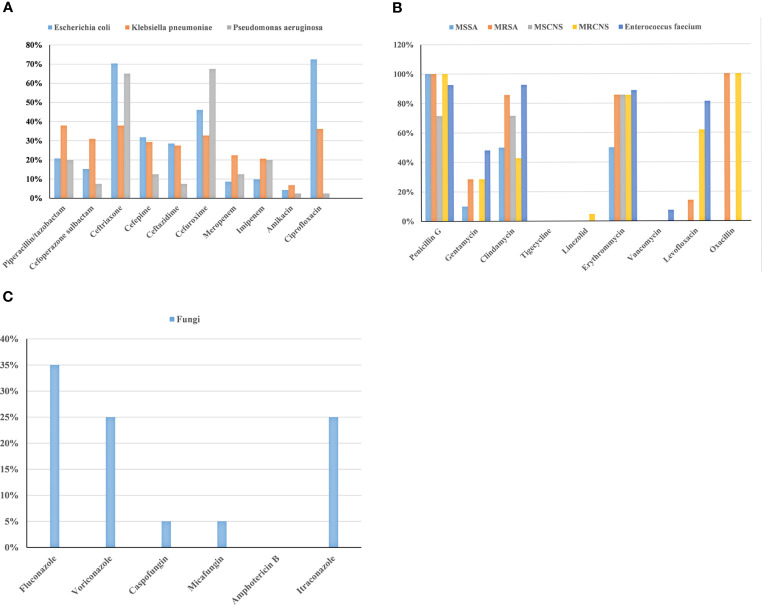
Analysis of drug-resistance in BSIs with gram-negative bacteria **(A)**, gram-positive bacteria **(B)**, and fungi **(C)** among patients with HMs.

The results of the drug sensitivity test for the common pathogens are exhibited in **Figure 2**. *Escherichia coli* had the lowest rate of antibiotic resistance to amikacin (4.3%), followed by meropenem (8.7%) and imipenem (9.8%). The rates of antibiotic resistance of *Klebsiella pneumoniae* to amikacin, imipenem, and meropenem were 6.8%, 20.6%, and 22.4%, respectively. *Pseudomonas aeruginosa* had the lowest rates of antibiotic resistance to amikacin and ciprofloxacin, which were 2.5%. MRSA had no antibiotic resistance to tigecycline, linezolid, and vancomycin. MRCNS was completely sensitive to tigecycline and vancomycin. *Enterococcus faecium* had no resistance to tigecycline and linezolid. Fungal resistance was low to caspofungin (5%) and micafungin (5%). Meanwhile, no fungus resistant to amphotericin B was found in this study.

### Establishment of a nomogram for predicting 30-day mortality after BSI

**Figure 3:**
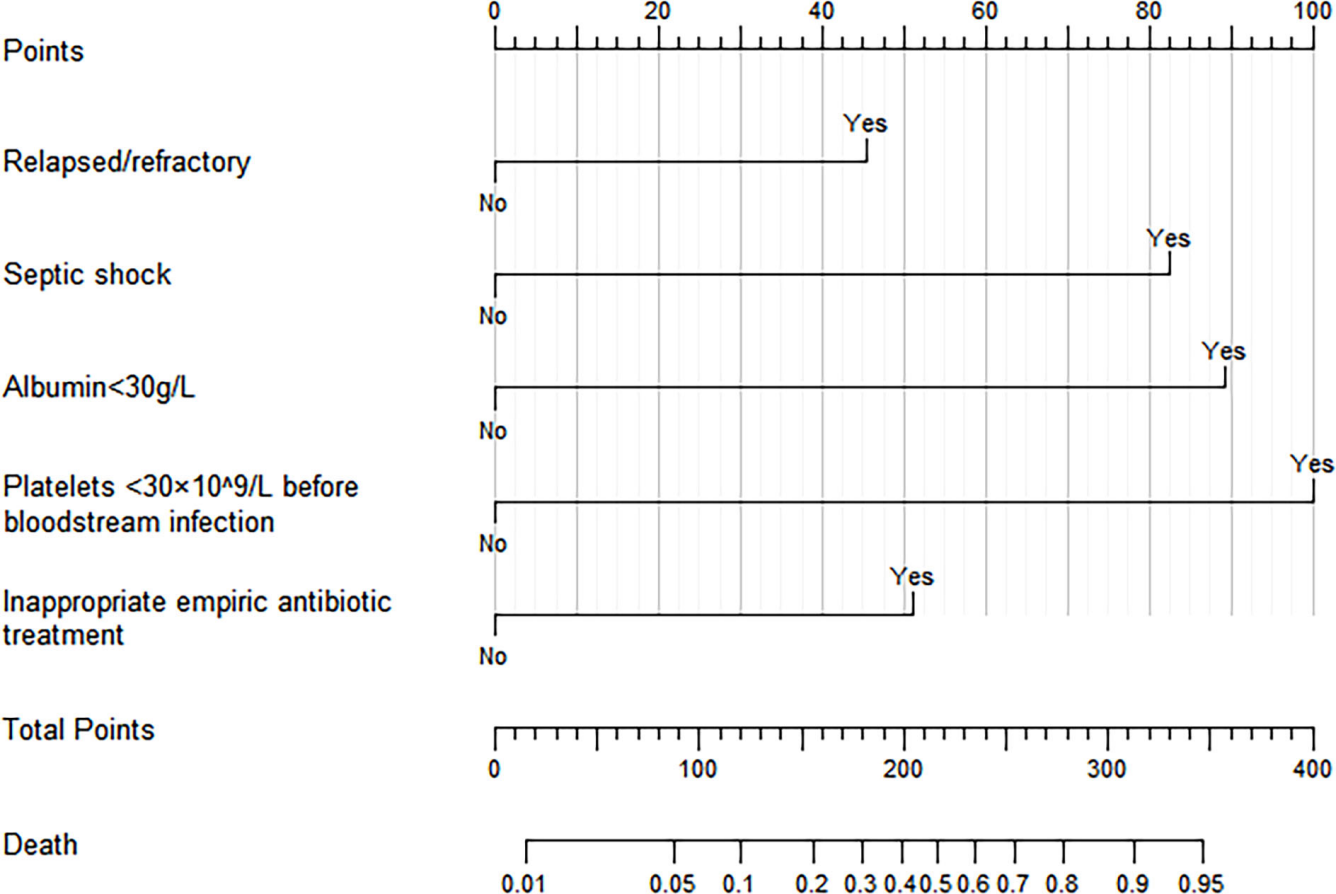
Nomogram for predicting 30-day mortality after BSIs in patients with HMs. (The “Yes” or “No” of each variable corresponded to the score on the “Points” axis, then the individual scores were added together to obtain the total score, the total score on the “Total points” axis corresponded to the dot of “Death” axis, which was the predicted probability of 30-day mortality).

In the training cohort, univariate logistic regression analysis showed that septic shock, relapsed/refractory HM, length of stay before BSI, concurrent pulmonary infection, concurrent perianal infection, neutropenia, albumin <30g/l, platelets <30×10^9^/l before BSI, platelets <30×10^9^/l after BSI, placement of the urinary catheter, secondary DIC, inappropriate empiric antibiotic treatment, combined empiric antibiotic treatment, and chemotherapy regimen containing glucocorticoid were associated with 30-day mortality after BSI (p<0.05 for all, **Table 2**). Further multivariate logistic regression analysis revealed that septic shock (p<0.001, OR=6.548, 95%CI: 3.042-14.092), relapsed/refractory HM (p=0.008, OR=2.811, 95%CI: 1.317-6.002), albumin <30g/l (P<0.001, OR=7.640, 95%CI: 3.529-16.542), platelets <30×10^9^/l before BSI (P<0.001, OR=9.761, 95%CI: 3.511-27.132), and inappropriate empiric antibiotic treatment (P=0.002, OR=3.213, 95%CI: 1.522-6.785) were independent risk factors for 30-day mortality after BSI in patients with HMs (**Table 2**). Based on the result of multivariate logistic regression analysis, we established a visual nomogram for predicting 30-day mortality after BSI in patients with HMs (**Figure 3**).

**Table 2:** Univariate and multivariate logistic regression analyses of 30-day mortality in the training cohort.

Univariate analysis			Multivariate analysis		
Characteristic	OR (95%CI)	*P* value	Characteristic	OR (95%CI)	*P* value
Male	1.759 (0.962, 3.216)	0.067	Male		
Age ≥60 years	0.728 (0.357, 1.487)	0.384	Age ≥60 years		
Pathogens	–	0.999	Pathogens		
Gram-negative bacteria			Gram-negative bacteria		
Gram-positive bacteria			Gram-positive bacteria		
Fungi			Fungi		
Septic shock	8.078 (4.248, 15.358)	**<0.001**	Septic shock	6.548 (3.042, 14.092)	**<0.001**
Relapsed/refractory HMs	1.979 (1.116, 3.511)	**0.02**	Relapsed/refractory HMs	2.811 (1.317, 6.002)	**0.008**
Hematopoietic stem cell transplantation	1.392 (0.532, 3.644)	0.501	Hematopoietic stem cell transplantation		
Nosocomial infection	1.951(0.712, 5.347)	0.194	Nosocomial infection		
Length of stay before BSI, days	1.017 (1.005, 1.030)	**0.007**	Length of stay before BSI, days		0.055
Concurrent pulmonary infection	3.858 (1.130, 13.177)	**0.031**	Concurrent pulmonary infection		0.238
Concurrent intestinal infection	0.938 (0.352, 2.500)	0.897	Concurrent intestinal infection		
Concurrent perianal infection	2.186 (1.022, 4.674)	**0.044**	Concurrent perianal infection		0.233
White blood cell count, 10^9^/l	0.955 (0.977, 1.012)	0.555	White blood cell count, 10^9^/l		
Neutropenia	2.569 (1.143, 5.774)	**0.022**	Neutropenia		0.190
Profound neutropenia	1.213 (0.674, 2.183)	0.519	Profound neutropenia		
Albumin <30g/l	5.691 (3.153, 10.272)	**<0.001**	Albumin <30g/l	7.640 (3.529, 16.542)	**<0.001**
PCT, ng/ml	1.010 (0.997, 1.022)	0.137	PCT, ng/ml		
CRP, mg/l	1.003 (1.000, 1.007)	0.055	CRP, mg/l		
Platelets <30×10^9^/l before BSI	6.422 (2.789, 14.788)	**<0.001**	Platelets <30×109/l before BSI	9.761 (3.511, 27.132)	**<0.001**
Platelets <30×10^9^/l after BSI	3.948 (1.701, 9.166)	**0.001**	Platelets <30×109/l after BSI		0.312
Placement of urinary catheter	3.729 (1.845, 7.537)	**<0.001**	Placement of urinary catheter		0.274
Placement of PICC/CVC	0.875 (0.506, 1.512)	0.632	Placement of PICC/CVC		
Secondary DIC	6.422 (2.789, 14.788)	**<0.001**	Secondary DIC		0.124
BSI with MDR bacteria	1.109 (0.638, 1.927)	0.714	BSI with MDR bacteria		
Inappropriate empiric antibiotic treatment	2.044 (1.163, 3.590)	**0.013**	Inappropriate empiric antibiotic treatment	3.213 (1.522, 6.785)	**0.002**
Combined empiric antibiotic treatment	3.136	**0.003**	Combined empiric antibiotic treatment		0.214
Chemotherapy regimen containing glucocorticoid	2.093 (1.202, 3.645)	**0.009**	Chemotherapy regimen containing glucocorticoid		0.410

The bold values means “p<0.05”. A p <0.05 was statistically significant.

### Calibration and validation of the nomogram

**Figure 4:**
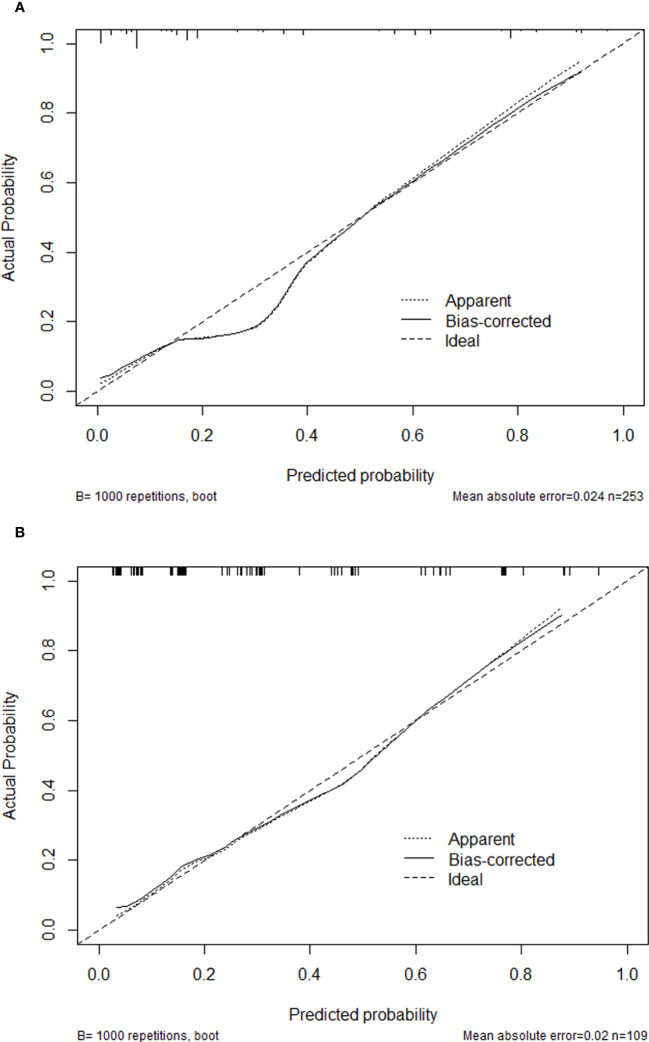
Calibration plots of the nomogram in the training cohort **(A)** and the validation cohort **(B)**.

**Figure 5:**
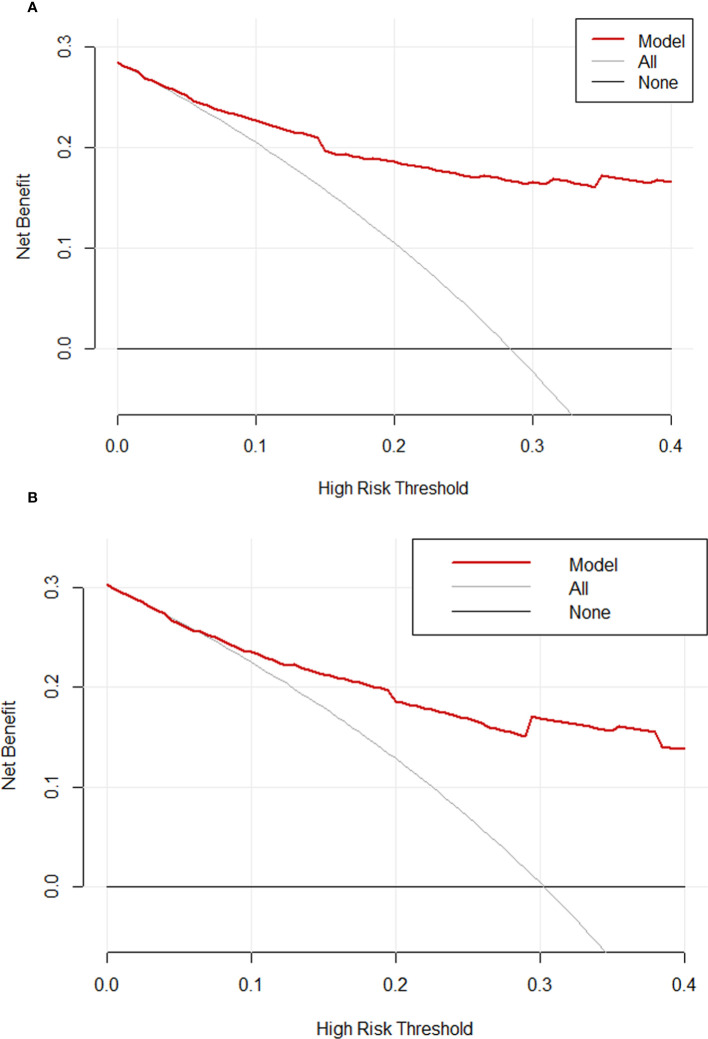
Decision curve analysis for the training cohort **(A)** and the validation cohort **(B)**.

The C-indexes of the nomogram were 0.870 (95%CI: 0.820-0.921) and 0.825 (95%CI: 0.737-0.912) in the training and validation cohorts, which indicated that the model had good discrimination. As shown in **Figure 4**, the calibration plots of the nomogram in both cohorts suggested that 30-day mortality after BSI predicted by the nomogram model was consistent with the actual mortality. Decision curves were used to evaluate the net clinical benefit of the nomogram model. The decision curves of the nomogram in the training and validation cohorts showed that the model for predicting 30-day mortality after BSI was more beneficial than all patients with BSIs or none with BSIs (**Figure 5**).

## Discussion

BSI is a common infection in patients with HMs. BSI negatively impacts patients with HMs, which may lead to death (Kochanek et al., 2019). Based on the complexity and high mortality of BSIs in HMs, we retrospectively analyzed the hospital data to identify pathogens’ distribution and antibiotic resistance. Meanwhile, we developed a nomogram for predicting the 30-day mortality of patients with BSIs in HMs. Our study represented a large-scale, single-center experience, and the results deepened our understanding of BSIs in patients with HMs.

Acute leukemia (acute myeloid leukemia and acute lymphocytic leukemia) was the most common HMs with BSIs in our study. The reason may be that patients with acute leukemia frequently received intensive chemotherapy and had long-term neutropenia (Garcia-Vidal et al., 2018). Neutropenia was often associated with life-threatening infections during cytotoxic chemotherapy (Kuo et al., 2017). Research showed that the rate of infections was 86.9% in patients with acute leukemia during the induction chemotherapy (Kato et al., 2018). Therefore, active infection control measures were necessary for patients with acute leukemia.

In the early days, researchers generally observed that gram-positive bacteria were the most common in BSIs (Ortega et al., 2005; Safdar et al., 2006; Huttunen et al., 2015). This may be associated with the prophylactic use of fluoroquinolone (Kern et al., 2018). However, this trend has reversed in recent years, and BSIs with gram-negative bacteria were more prevalent than gram-positive bacteria (Chen et al., 2017; Mimura et al., 2020; Haddad et al., 2021). Our study also confirmed the changing trend. We collected 362 pathogens, mainly gram-negative bacteria (70.4%), the most common being *Escherichia coli*. A retrospective study showed that BSIs with gram-negative bacteria accounted for 65% from 2007 to 2017, of which *Escherichia coli* was the most common (Haddad et al., 2021). A study from China suggested that gram-negative bacteria accounted for 64.7%, gram-positive bacteria accounted for 27.7%, and fungi accounted for 7.7% of BSIs in patients with HMs (Chen et al., 2021). In the present study, BSIs with gram-positive bacteria accounted for 24%, and coagulase-negative staphylococci were the most common, similar to other reports (Tang et al., 2018; Amanati et al., 2021). Most BSIs with gram-positive bacteria may be caused by long-term or repeated placement of PICC and CVC (van den Bosch et al., 2021). Candida was the primary pathogen of fungal BSIs, but the positive rate of fungal blood culture was low. This may be related to the difficulty of fungal culture and the lack of specific detection methods (Pappas et al., 2018). Therefore, blood cultures should be drawn actively and repeated for patients with fever if necessary. Moreover, new detection methods, such as second-generation sequencing, help detect the fungus (Peri et al., 2022). When patients have a poor response to antibiotics, they should be alert to fungal infections and be treated with antifungal agents as soon as necessary (Freifeld et al., 2011). The distribution of pathogens in our study was distinct from a study from Spain (Garcia-Vidal et al., 2018). This may be due to differences in region and habits of medication.

With the extensive use of antibiotics, bacterial drug resistance is becoming more serious. The mechanisms of bacterial resistance include the production of inactive enzymes and modifying enzymes, the change of target sites, the expression of efflux pumps, and the reduced permeability of the bacterial outer membrane (Blair et al., 2015). Identifying patterns of local bacterial resistance is a prerequisite for selecting appropriate antibiotics. Our study found that *Escherichia coli* and *Klebsiella pneumoniae* were sensitive to carbapenem antibiotics and amikacin, and *Pseudomonas aeruginosa* was sensitive to amikacin. This result was consistent with other studies (Trecarichi et al., 2015; Zhao et al., 2020; Shi et al., 2022). The antibiotics above may be appropriate for the common BSIs with gram-negative bacteria in clinical practice. Compared to our study showing a low resistance rate of *Pseudomonas aeruginosa* to ciprofloxacin (2.5%), other studies have shown various resistance in *Pseudomonas aeruginosa* to ciprofloxacin (2.4%-80.3%) (Sligl et al., 2015; Trecarichi et al., 2015; Chen et al., 2017; Zhao et al., 2020). The inconsistent results among the studies may be due to regional differences and sample sizes. Common gram-positive bacteria were susceptible to tigecycline, linezolid, and vancomycin. In the present study, MRSA, MRCNS, and vancomycin-resistant *Enterococcus faecium* were also detected (Haddad et al., 2021). With the application of antibiotics, the emergence of extended-spectrum β-lactamase-producing bacteria and MDR bacteria has become one of the thorniest problems. Therefore, it is necessary to have clear indications for the use of antibiotics. At the same time, the use of antibiotics must be cautious, and the distribution of local pathogens and drug-resistance results should be considered. Fungal infections were not uncommon in patients with HMs. In our hospital, *Candida tropicalis* was the most common. Amphotericin B, caspofungin, and micafungin were effective for patients with fungal BSIs in HMs. Xiao et al. found that the mortality of patients with fungal BSIs in HMs was 40.5%, much higher than that of patients with common fungal infections in HMs (Xiao et al., 2020). Therefore, it is vital to understand fungi’s distribution and drug resistance of fungi for treating fungal BSIs.

BSI was a common complication associated with high mortality in a patient with HM. The 30-day mortality was 29% in our study population, and other studies reported mortality ranging from 10% to 32% (Nørgaard et al., 2006; Åttman et al., 2015; Tang et al., 2018; Chen et al., 2021). Some studies about the risk factors for the poor prognosis of patients with BSIs in HMs were published (Tumbarello et al., 2012; Trecarichi et al., 2016; Tang et al., 2018; Shi et al., 2022). Nomograms are commonly used to evaluate the prognosis of tumor patients (Balachandran et al., 2015). We developed a nomogram for predicting the 30-day mortality of patients with BSIs in HMs. According to the evaluation with the c-indexes, calibration plots, and decision curves, the nomogram had satisfactory discrimination, consistency, and clinical benefit. The nomogram showed that septic shock, relapsed/refractory HM, albumin <30g/l, platelets <30×10^9^/l before BSI, and inappropriate empiric antibiotic treatment were independent risk factors for 30-day mortality after BSI in patients with HMs.

A notable finding in our study was the identification of a previously unnoted risk factor for 30-day mortality, namely platelets <30×10^9^/l before BSI. Patients with HMs developed thrombocytopenia due to myelosuppression. After BSI, especially progression to sepsis, platelet levels in patients with HMs may decrease further (Ghimire et al., 2021). Platelets are essential in primary hemostasis and are also involved in angiogenesis, tissue repair, and inflammation (Vinholt, 2019). Menard et al. found that thrombocytopenia was associated with major bleeding events and increased mortality in patients with septic shock (Menard et al., 2019). Thus, patients with BSIs in HMs with platelets less than 30×10^9^/l had a higher risk of death at 30 days, possibly because they were more prone to major bleeding. Therefore, attention should be paid to the patients with BSIs in HMs who had low platelet levels, and appropriate platelet transfusion should be given to improve the prognosis of patients.

Septic shock is characterized by systemic hypoperfusion, often leading to organ failure and high mortality (Cecconi et al., 2018). Our study and others confirmed that septic shock was an independent risk factor for death in patients with HMs after BSI (Criscuolo et al., 2019; Chen et al., 2020; Royo-Cebrecos et al., 2022). Patients with low oxygen saturation, hypotension, or disturbance of consciousness need to be identified early and intervened as soon as possible, including fluid resuscitation and vasopressors. The association between the state of HM and mortality had been previously determined (Tang et al., 2018; Chen et al., 2021). We also found that relapsed/refractory HM was an independent risk factor for 30-day mortality after BSI in patients with HMs. Albumin is synthesized by the liver and reflects liver function and nutritional status. The study by Tang et al. suggested that albumin <30g/l was closely related to 30-day mortality in patients with HMs and BSIs (Tang et al., 2018). The same finding was confirmed in our study. Monitoring the albumin level and, if necessary, infusion of albumin is of great significance for the prognosis of patients with BSIs in HMs.

Empiric antibiotic therapy is one of the primary measures of anti-infection treatment. For patients with BSIs in HMs, the use of antibiotics is best based on the drug sensitivity, but it takes a while to wait for the blood culture results. The infection may further aggravate or even become life-threatening if antibiotics are not given in time. Previous studies have shown that inappropriate empiric antibiotic therapy was a risk factor for mortality in patients with BSIs among HMs (Tang et al., 2018; Tang et al., 2020; Zhao et al., 2022). This was also confirmed in the present study. Empiric antibiotic therapy for patients with BSIs in HMs should be individualized, considering previous antibiotic use, current epidemiology, and appropriate guidelines (Freifeld et al., 2011; Chinese Society of Hematology et al., 2020). Other factors such as neutropenia, age, placement of the urinary catheter, and nosocomial infection were not ultimately included in the nomogram in our study, possibly because of differences in the population and region and the retrospective nature of the study (Tumbarello et al., 2012; Tang et al., 2018; Shi et al., 2022).

Our study has some limitations. First, our study is retrospective, with limited samples and biases, and we only conducted internal validation. External validation and prospective studies are needed to optimize our model. Second, our study was based on diagnostic, laboratory testing, and therapeutic regimens at a single center, and there may be regional differences in findings.

In conclusion, our study suggested that gram-negative bacterial BSIs were predominant in patients with HMs. Meanwhile, we found that septic shock, relapsed/refractory HM, albumin <30g/l, platelets <30×10^9^/l before BSI, and inappropriate empiric antibiotic treatment were independent risk factors for 30-day mortality after BSI in patients with HMs. Based on these factors, the nomogram was developed and validated to have good predictive ability. It can be used to evaluate the prognosis of patients promptly for clinicians.

## Data availability statement

The original contributions presented in the study are included in the article/supplementary material. Further inquiries can be directed to the corresponding author.

## Ethics statement

West China Hospital of Sichuan University Ethics Committee approved this study (2022-1930).

## Author contributions

JW collected clinical data, interpreted the results, wrote, and revised the manuscript. MW and AZ participated in collecting data, data statistics. HZ and MM revised the manuscript. XL checked statistical methods. TN participated in the study design and revised the manuscript. All authors contributed to the article and approved the submitted version.
